# Analysis of Water Fluoridation Debates on Meta Platforms Using Advanced Machine Learning Approaches

**DOI:** 10.1177/23800844251369336

**Published:** 2025-09-15

**Authors:** N. Torwane, R. Lalloo, D. Ha, L. Do

**Affiliations:** 1 The University of Queensland, School of Dentistry, Brisbane, QLD, Australia

**Keywords:** health communication, public opinion, social media, preventive dentistry, natural language processing, Sentiment Analysis

## Abstract

Social media platforms, such as Facebook and Instagram, play a pivotal role in shaping public opinion on health interventions, including community water fluoridation (CWF). Despite its recognition as a safe and effective public health measure, CWF remains a polarizing topic, with misinformation on these platforms contributing to public mistrust. This study collected 109,117 Facebook and Instagram posts from 2014 to 2023 to examine public sentiment surrounding CWF. The analysis revealed a mix of opinions, with 42.1% positive, 39.1% negative, and 18.8% neutral sentiments. Trends highlighted a surge in negative sentiment during 2017–2019, likely influenced by misinformation and significant public events, while positive sentiment has gradually regained ground in recent years. Key themes included health benefits, safety concerns, and government trust, with positive discussions emphasizing CWF’s role in public health and negative discussions focusing on risks and chemical exposure. The study employed advanced sentiment analysis models to highlight the importance of monitoring public discourse and addressing misinformation, thereby promoting trust and support for evidence-based health policies, such as CWF. These findings offer digital data-driven insights for public health communication strategies, enhancing community understanding and acceptance of vital health interventions.

**Knowledge Transfer Statement:**

The findings of this study can inform public health agencies and policymakers about public concerns and support for water fluoridation. By identifying sentiment trends and misinformation on social media, communication strategies can be tailored to improve public understanding and acceptance of fluoridation as a preventive health measure.

## Introduction

In today’s digital age, social media platforms have become central to shaping public opinion on health, social, and political issues (Cinelli et al. 2020). Community water fluoridation (CWF)—the adjustment of fluoride levels in public water supplies to prevent dental caries—is one such topic that frequently surfaces in online discussions. Despite being widely endorsed by health authorities such as the World Health Organization (WHO), the Centers for Disease Control and Prevention (CDC), and Australia’s National Health and Medical Research Council (NHMRC), CWF remains polarizing, partly due to the dissemination of misinformation on social media platforms (Lotto et al. 2022; Mertz and Allukian 2014).

Social media offers several advantages as a data source for public health research. With billions of active users, platforms such as Facebook and Instagram generate a vast volume of user-generated content that reflects diverse public attitudes (Black et al. 2015; Statista Research Department 2024). These networks promote digital communities where people share personal experiences, opinions, and even unverified claims about health interventions (Gabarron and Wynn 2016). For example, misinformation about vaccines and alternative health remedies (e.g., amber necklaces for teething) has been widely circulated, influencing health behaviors and fueling skepticism (George et al. 2013; Larson et al. 2016). This pattern is also evident in CWF debates, in which anecdotal concerns and mistrust often overshadow peer-reviewed evidence (Meier et al. 2007; Helmi et al. 2018; Mackert et al. 2021).

While social media enables real-time sentiment tracking and broader engagement than traditional surveys do, it also introduces important limitations. Not all population groups are equally active online, and emotionally charged or controversial posts tend to attract disproportionate attention, potentially skewing sentiment analyses toward more polarized views (Cavazos-Rehg et al. 2015; Vosoughi et al. 2018; Eysenbach 2021). This introduces a risk of overrepresenting extreme opinions, which may not accurately reflect broader community sentiment. These limitations must be acknowledged when interpreting social media data in public health contexts.

Hashtag trends and user interactions can provide indirect insight into how health information circulates online. However, strong assertions—such as the role of recommendation systems in amplifying specific content—must be made cautiously. Although algorithmic promotion is well documented in other domains (Cinelli et al. 2020), more empirical evidence is needed to understand its specific role in debates surrounding CWF.

This study aimed to conduct a detailed sentiment analysis of user-generated content from Facebook and Instagram to examine public debate surrounding CWF. By identifying the key sentiment patterns, misinformation themes, and engagement trends over time, the study contributes to a growing body of work on digital health surveillance. These insights are intended to guide public health communication strategies and support evidence-based policymaking.

## Materials and Methods

### Scope of the Study

This study used sentiment analysis to investigate public perceptions of CWF using content from 2 meta platforms: Facebook and Instagram. Posts were collected from January 2014 to December 2023, providing a 10-y longitudinal dataset to track changes in sentiment and engagement patterns. These platforms were selected due to their extensive user bases, comprehensive content-sharing infrastructure, and influence in shaping health communication and public discourse (Chou et al. 2018; Oh et al. 2020; Umunyana et al. 2024).

Facebook and Instagram were analyzed together because they operate under the same corporate entity, share similar content algorithms, and allow for cross-posting and unified data management. Merging data from both platforms ensured consistency in data structure and allowed the analysis to capture broader interactions and public discussions about CWF. This approach also aligns with prior social media research methodologies, in which combining platform data enhances the validity of sentiment trends and engagement patterns (Chou et al. 2018; Boon-Itt and Skunkan 2020).

### Ethical Approval and Data Confidentiality

The study exclusively used publicly available data from the Meta Platform. As the project involved no human subjects or identifiable private information, it received ethical exemption from the University of Queensland’s Human Ethics Committee (approval No. 2022/HE002248). All collected data were encrypted and password protected to ensure secure storage. Personally identifiable information, such as usernames or direct identifiers, was automatically anonymized using a unique identifier system before processing. To further protect user privacy, only relevant data for the sentiment analysis was retained, and unnecessary metadata or nontextual content was removed. These measures ensured compliance with the ethical guidelines for the use of secondary data in digital health research.

### Sentiment Analysis Workflow

The sentiment analysis workflow consisted of 6 primary stages: data collection, search strategy, data processing, sentiment scoring, model validation, and performance evaluation. This workflow outlines a structured framework for conducting sentiment analysis as a task within natural language processing (NLP), rather than implying sentiment analysis as a procedure itself.

#### Data collection and source identification

Data were extracted using Meta’s Graph API, which provides access to public posts and comments on Facebook and Instagram. The API enabled the structured extraction of post text and associated metadata, including the time of publication, likes, shares, and other engagement indicators. The dataset initially comprised 109,117 posts, which were then filtered and refined for analysis.

Geolocation data were not included in this analysis. While Meta’s Graph API provides some metadata fields, it does not reliably support post-level geographic tagging for public posts due to privacy restrictions and inconsistent user-provided location data. Consequently, this study did not apply geographic filtering to identify the country, city, or region of origin. This limits our ability to associate sentiment trends with specific jurisdictions or policy environments. Our focus was on overall sentiment trends rather than region-specific responses.

#### Search strategy and keyword filters

To ensure the relevance of posts to CWF, specific search terms were predefined. These included popular hashtags and keywords such as “#WaterFluoridation,” “#CommunityWaterFluoridation,” “#FluorideInWater,” and variations of these terms. Hashtags (e.g., #FluorideFree, #SayNoToFluoride) served as user-generated topical markers, while keywords (e.g., “fluoride,” “toxic water,” “fluoridation harms”) were unstructured search terms embedded in natural language. This dual strategy allowed for broader capture of relevant content and aligns with best practices in social media mining for health communication. These keywords were selected based on prior studies of CWF-related online discourse (Oh et al. 2020; Boon-Itt and Skunkan 2020). The search was restricted to posts written in English, as the sentiment analysis tools used in the study were optimized for English-language processing.

#### Data preprocessing and cleaning

After extraction, the dataset underwent a thorough data-cleansing process using Python libraries. The preprocessing phase involved removing duplicates, hyperlinks, emojis, special characters, punctuation, user mentions, and stopwords. Posts with fewer than 5 words were excluded to avoid processing extremely short or ambiguous content. This standardization step was essential to prepare the data for NLP tasks. The cleaned dataset was then stored in extensible markup language (XML) format, which supported structured parsing and reproducibility of the analysis.

#### Sentiment classification framework

For sentiment scoring, we used the SentimentIntensityAnalyzer module from the Natural Language Toolkit (NLTK) in Python. This lexicon- and rule-based model is particularly effective for analyzing the short, informal, and emotion-rich language typical of social media posts (George et al. 2013). NLTK assigns a sentiment score ranging from −1 (highly negative) to +1 (highly positive), with 0 denoting a neutral tone. Each post was categorized into positive, negative, or neutral sentiment categories.

#### Model validation and reliability testing

To validate the sentiment labels, we randomly selected a subset of posts (*n* = 1,000) and manually annotated them for ground-truth comparison. Model accuracy was calculated based on the agreement between automated and manual classifications. Discrepancies were reviewed and used to fine-tune preprocessing settings and sentiment thresholds. This ensured reliability and improved alignment between machine-generated sentiment labels and human interpretation (Oh et al. 2020).

### Reporting Guidelines

This study adheres to the STROBE (Strengthening the Reporting of Observational Studies in Epidemiology) guidelines for observational research (Appendix File 3).

## Factor-Based Data Analysis

### Engagement Metrics Analysis

To evaluate public interaction with CWF content, we analyzed engagement metrics such as likes, shares, and comments. These metrics were recorded and correlated with key public health announcements or fluoridation debates. Changes in engagement volume were tracked to determine fluctuations in public interest and responsiveness to CWF-related discussions (Chou et al. 2018).

### Sentiment Trends and Thematic Insights

We mapped sentiment patterns year by year to observe shifts in public opinion. Sudden spikes in negative sentiment were examined in context with concurrent misinformation events or health policy changes. This temporal mapping enabled event-driven sentiment analysis and the identification of potential misinformation surges.

### Word Sentiment and Frequency Analysis

We applied a lexicon of pretagged positive and negative terms to identify the most frequently used emotion-rich words. The frequency of each term was normalized by the total number of posts per year to facilitate trend comparison. This helped identify terms that shaped public sentiment and provided insights into the themes of public discussions (Boon-Itt and Skunkan 2020).

### Co-occurrence Network Analysis

We constructed a network graph using high-frequency keywords that co-occurred in CWF-related posts. The co-occurrence matrix revealed clusters of related themes, such as health, safety, toxicity, and trust, and their interrelationships. This technique illuminated the central narratives in CWF discussions and the terms most likely to trigger positive or negative sentiment (Mertz and Allukian 2014; Helmi et al. 2018).

### Machine Learning–Based Sentiment Model Evaluation

We applied 4 commonly used text classification algorithms: logistic regression, decision tree, naïve Bayes, and random forest. Model performance was assessed using standard metrics (James et al. 2013; Umunyana et al. 2024):

- Accuracy: Correct predictions out of all predictions
- Precision: Correct positive predictions among predicted positives
- Recall: True positives captured from all actual positives
- F1 score: Harmonic mean of precision and recall
- Area under the curve (AUC): model’s ability to distinguish sentiment categories

Logistic regression achieved the highest accuracy and AUC, making it the most effective model for this dataset. The ensemble model (random forest) also demonstrated strong performance, while naïve Bayes showed lower reliability in handling complex sentence structures.

## Results

### Engagement Metrics Analyses

Between 2014 and 2023, 109,117 posts related to CWF discussions were collected from Meta platforms (Facebook and Instagram). Facebook contributed 73,938 (67.8%) posts, and Instagram provided 35,179 (32.2%). After data processing to remove duplicates, URL links, images, and other irrelevant content, 63,806 (58.5%) posts from Facebook and Instagram were retained for analysis.

**Figure 1.**
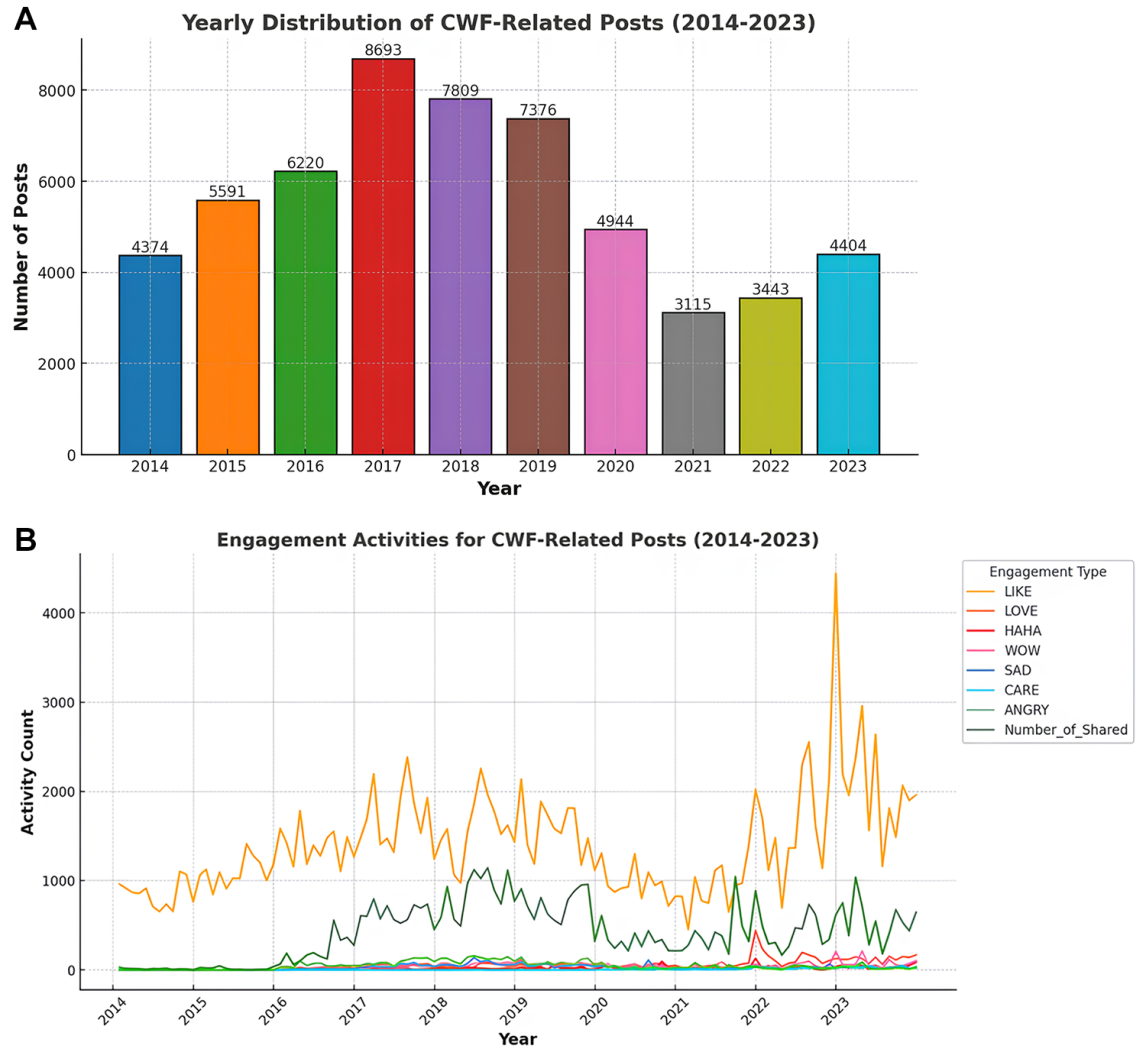
(**A**) Yearly distribution of community water fluoridation (CWF)–related posts. This bar chart displays the yearly volume of CWF-related posts on Meta platforms (Facebook and Instagram) from 2014 to 2023. The data show a steady increase in post volume from 2014, peaking in 2017 (8,693 posts), followed by a gradual decline from 2021 onward. The lowest engagement was recorded in 2021 (3,115 posts), with a slight increase in activity in 2023 (4,404 posts). (**B)** Engagement activities for CWF-related posts. This line graph illustrates engagement activities (likes, shares, and other reactions) related to CWF posts on Meta platforms (Facebook and Instagram) from 2014 to 2023. The data show a steady rise in engagement over time, with like and share activities being the most dominant interactions. A notable spike in engagement is observed in 2022 to 2023, particularly in likes, indicating increased public interaction with CWF-related content during this period.

The post count analysis showed fluctuations in public engagement over time (Fig. 1A). From 2014 to 2017, post volume rose steadily, indicating growing interest in CWF, with peak engagement occurring between 2017 and 2019. Post activity declined gradually from 2020 onward, with moderate engagement levels of 3,000 to 5,000 posts from 2020 to 2023, suggesting a gradual decrease in public interest.

Engagement patterns revealed that the “like” reaction was most common, peaking in 2022, with a rise in “share” reactions, indicating users preferred likes and shares over other forms of interaction (Fig. 1B). Regarding post content, 42,789 (67.1%) posts contained sentences ranging from 100 to 300 characters, while 3,276 (5.1%) posts exceeded 500 characters, reflecting the social media preference for concise, easily digestible content (Appendix File 1).

### Textual Sentiment Analyses

#### Sentiment trends and patterns

Of the posts regarding CWF, 42.1% conveyed a positive sentiment, 39.1% were negative, and 18.8% were neutral, indicating a mix of public support and opposition.

Sentiment trends fluctuated notably over time. From 2014 to 2016, public opinion remained stable, with positive sentiments consistently outnumbering negative ones. In 2016, among 6,220 posts, 49.1% were positive, while 28.9% were negative, indicating a generally favorable stance on CWF.

**Figure 2.**
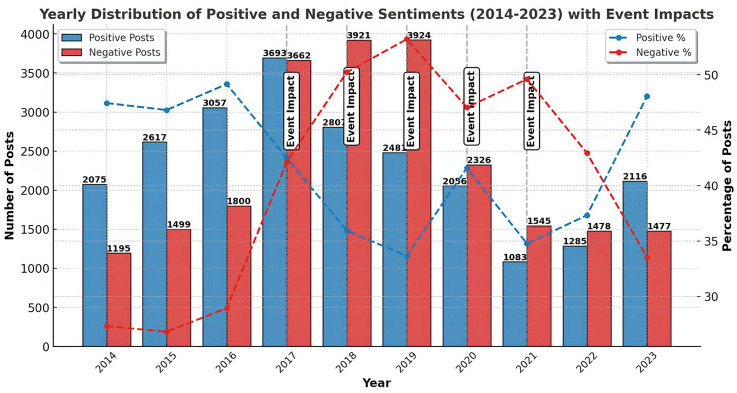
Sentiment trends in community water fluoridation (CWF)–related posts over time. This chart illustrates the yearly distribution of positive and negative sentiments in CWF-related posts from 2014 to 2023. The bars represent the number of positive and negative posts per year, while the dashed lines indicate the percentage trends of each sentiment. The data show a significant increase in negative sentiment from 2017 to 2019, aligning with key events marked as “Event Impact.” After 2019, both positive and negative sentiments declined, with negative sentiment leading slightly during 2020 to 2021. However, in 2022 to 2023, positive sentiment rebounded, suggesting a shift toward a more balanced public perception of CWF.

Negative sentiment spiked in 2017, peaking in 2018 when negative posts significantly outnumbered positive ones, largely due to adverse events (Fig. 2). By 2019, sentiment had shifted further, with 53.2% of posts being negative and 33.6% positive out of 7,376 posts, reflecting a rise in controversy and misinformation.

From 2020 to 2021, both positive and negative sentiments declined, although negative sentiment remained dominant, accounting for 47.0% and 49.6% of posts, respectively. Event markers suggest that discussions during this period were reignited by policy debates or misinformation. By 2022–2023, sentiment balance improved, with positive perceptions increasing. In 2023, among 4,404 posts, 48.0% were positive while 33.5% were negative, signaling a more favorable outlook. However, engagement remained lower than in peak years, suggesting waning public interest in the topic.

Overall, public discussions on CWF have been mostly positive, although negative sentiments were slightly more prominent during 2017 to 2019. However, the intensity of negative reactions has recently declined while positive sentiments have gradually increased.

### Word Sentiment Analysis

**Figure 3.**
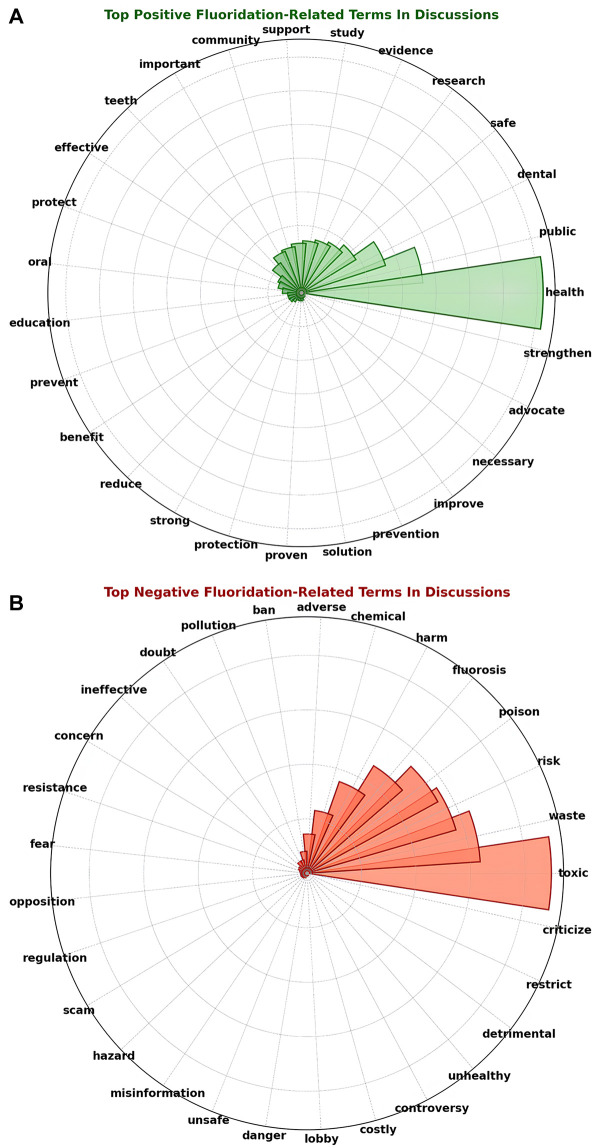
(**A**) Top positive (favorable) words in community water fluoridation (CWF)–related posts. This radial plot visualizes the most frequently used positive terms in CWF-related posts on Meta platforms. Prominent terms such as “health,” “public,” and “strengthen” emphasize the perceived health benefits, societal advantages, and advocacy for CWF. (**B**) Top negative (unfavorable) words in CWF-related posts. This radial plot highlights the most frequently used negative terms in CWF-related posts on Meta platforms. Words such as “toxic,” “harm,” and “chemical” underscore safety concerns and skepticism surrounding CWF, reflecting public apprehensions about its risks and effects.

The analysis of favorable terms in posts about CWF on Meta platforms suggests social acceptance of fluoridation (Fig. 3A). The most common term, “health,” reflects the public’s association of fluoridation with health benefits. Other frequently used terms include “public,” “strengthen,” and “advocate,” indicating a focus on CWF’s broader societal health advantages. Words like “safe,” “research,” and “evidence” highlight scientific backing and safety, while terms such as “protection,” “prevent,” and “proven” convey its effectiveness as a preventive health measure. Additional terms, such as “community,” “education,” and “support,” emphasize the importance of community benefits and educational advocacy.

In contrast, negative terms used in posts expressing unfavorable views illustrate different concerns (Fig. 3B). The term “toxic” is the most common, indicating fears of harmful effects from fluoridation. Other key terms, such as “criticize,” “waste,” and “fluorosis,” reflect concerns about health risks, potential mismanagement, and side effects such as dental fluorosis. Terms such as “chemical,” “harm,” and “poison” reveal safety concerns related to chemical exposure, while “adverse,” “unsafe,” and “detrimental” express fears of adverse health impacts. In addition, words such as “controversy,” “lobby,” and “misinformation” suggest public distrust in fluoridation practices and information.

Overall, positive discussions focus on health, safety, and community benefits, while negative discussions focus on health risks, chemical safety concerns, and policy criticisms.

#### Word co-occurrence network analysis

**Figure 4.**
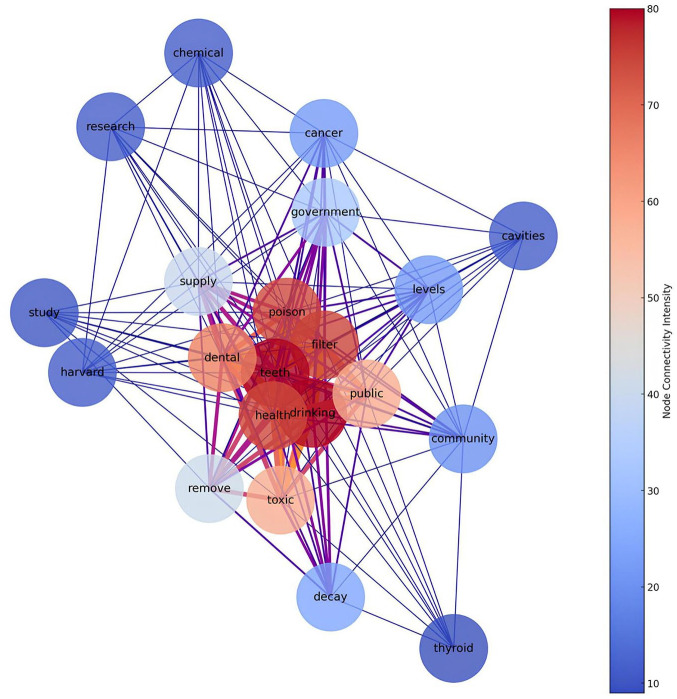
Word co-occurrence network analysis of community water fluoridation (CWF) discussions on Meta platforms. This network graph shows the relationships between frequently used terms in CWF-related posts. Nodes represent key terms, with their size and color intensity indicating frequency and connectivity. Central terms such as “health,” “drinking,” and “poison” dominate the discussion, highlighting concerns about dental health, water safety, and chemical risks. Peripheral nodes, such as “thyroid” and “community,” represent additional discussion themes, showcasing the multifaceted nature of public discourse on CWF.

The network graph displays prominent terms and their interconnections in CWF discussions on Meta platforms. Node size and color intensity indicate term frequency and centrality, with darker red nodes representing more frequent and strongly connected terms. The analysis reveals several main clusters in the discussion themes (Fig. 4).

Central terms, including “teeth,” “drinking,” “health,” and “poison,” are highly interconnected, suggesting that concerns about health risks related to drinking water and dental health dominate the conversation. “Filter” and “toxic” are also closely linked with these core terms, underscoring public apprehension about contamination and the need for water filtration.

Other prominent terms such as “chemical,” “government,” “cancer,” and “public” connect with these central themes, highlighting distrust in government actions on fluoridation and fears of severe health effects, such as cancer. Terms such as “research” and “Harvard” appear, indicating that academic studies from respected institutions are referenced in discussions.

On the periphery, terms such as “cavities,” “levels,” “community,” and “thyroid” reflect additional concerns, particularly around dosage levels, thyroid impacts, and community health outcomes.

In summary, the network graph shows that CWF discussions focus on health risks, drinking water concerns, and chemical safety, with frequent mentions of filtration and toxicity. Government trust and research are significant topics, reflecting public skepticism and ongoing debate.

### Model Performance Evaluation

Table 1 summarizes the performance of 4 supervised machine learning models used for sentiment classification. Logistic regression emerged as the top-performing model, achieving the highest accuracy (92.3%) and a substantial AUC value of 0.95. It also showed balanced performance across precision (92.35%), recall (92.31%), and F1 score (92.32%), indicating consistent classification ability across sentiment classes. Random forest followed with an accuracy of 88.5% and an AUC of 0.90, also demonstrating robust precision, recall, and F1 score values. The decision tree model yielded moderate results, with an accuracy of 87.0% and AUC of 0.87, while maintaining acceptable performance across other metrics. Naïve Bayes recorded the lowest accuracy (74.9%) and AUC (0.78), with relatively lower F1 score (72.61%), highlighting its limitations in handling sentiment classification in this context. The inclusion of these extended metrics enables a more comprehensive comparison across models, reflecting their strengths and limitations in processing informal, health-related social media content.

**Table 1.** Model Performance and Evaluation Details.

Model	Accuracy (%)	Precision (%)	Recall (%)	F1 Score (%)	AUC Value
Logistic regression	92.31	92.35	92.31	92.32	0.95
Decision tree	87.02	87.06	87.02	87.03	0.87
Naïve Bayes	74.85	76.63	74.85	72.61	0.78
Random forest	88.46	88.52	88.46	88.47	0.90

The table presents the performance metrics of various machine learning models used for the sentiment analysis of community water fluoridation–related posts. Logistic regression achieved the highest accuracy (92.31%) and area under the curve (AUC) value (0.95), making it the most effective model for this study. Other models, including Random forest, decision tree, and naïve Bayes, demonstrated varying performance levels, highlighting the importance of model selection in text classification tasks.

## Discussion

This study examined public discussions about CWF on Meta platforms, with a focus on how scientific communication, policy developments, and information sharing influenced public sentiment. The results suggest that social media plays a key role in shaping perceptions of preventive health measures. Similar influences have been seen in public conversations about vaccinations, where sentiment is shaped by exposure to misinformation, media coverage, and policy debates (Helmi et al. 2018; Vosoughi et al. 2018; Nguyen et al. 2022; Lotto et al. 2022).

Sentiment analysis revealed a polarized public discourse: 42.1% of posts were positive, 39.1% negative, and 18.8% neutral. These findings indicate that while there is clear support for CWF, concerns about safety, government trust, and misinformation continue to generate opposition. The increase in negative sentiment from 2017 to 2019 corresponds with significant policy developments and a rise in online misinformation. This trend aligns with earlier research on vaccine hesitancy during prominent antivaccine campaigns (Mertz and Allukian 2014; Chou et al. 2018; Helmi et al. 2018; Lotto et al. 2022). The Calgary case, in which online narratives influenced the cessation of fluoride, reflects the potential of digital misinformation to affect local policy (Canadian Academy of Health Sciences 2014; McLaren et al. 2016; O’Neill et al. 2019; City of Calgary 2021).

Neutral sentiment was also noteworthy. These discussions often reflected uncertainty or limited access to clear information. Previous studies suggest that neutral perceptions are more likely to shift in response to future information exposure, whether factual or inaccurate (McLaren and Singhal 2016; Helmi et al. 2018; Taddi et al. 2024). This highlights the need for clear, transparent, and inclusive communication strategies, especially for audiences not yet polarized. Iheozor-Ejiofor et al. (2024) confirmed in the Cochrane Review that CWF remains effective, although the effect may be smaller than previously estimated due to the broader availability of fluoride sources. However, its relevance remains high in areas with limited access to alternative fluoride delivery. These insights suggest that neutral sentiment groups may be exceptionally responsive to equity-based public health messaging.

Negative sentiment often featured emotionally charged language, including terms such as “toxic,” “harmful,” and “chemical,” which may reflect broader skepticism about chemically mediated health interventions (Gabarron and Wynn 2016; Roozenbeek and van der Linden 2020). This language resembles that seen in other public health debates, including those related to vaccines, and may contribute to heightened concern when left unchallenged (Chou et al. 2018). Research indicates that misinformation tends to spread faster than factual content does, particularly when it is emotionally engaging (Vosoughi et al. 2018; Boon-Itt and Skunkan 2020). In such environments, early and credible responses may be essential to avoid the entrenchment of misinformation (Helmi et al. 2018; Lotto et al. 2022).

Recent studies support the value of toxicity detection for understanding online health conversations. Hong et al. (2023) and Su and Thakur (2025) have shown that toxic or emotionally intense language is often associated with misinformation. Integrating toxicity detection with sentiment analysis could enhance the ability to interpret polarized or emotionally charged topics such as CWF.

In terms of model performance, logistic regression achieved the highest accuracy (92.3%) and AUC (0.9), followed closely by random forest (88.4%, AUC 0.9). Naïve Bayes demonstrated lower performance, with an accuracy of 74.8% and an AUC of 0.7. These results suggest that both logistic regression and ensemble models may be helpful in real-time tracking of public sentiment and early detection of misinformation trends (Quinlan 1986; Breiman 2001; Boon-Itt and Skunkan 2020).

The VADER tool was selected for its accessibility and ability to handle informal, short-form content commonly found on social media (Hutto and Gilbert 2014; Cheng et al. 2017). However, as a rule-based model, VADER lacks contextual understanding and may not recognize sarcasm or emotionally layered expressions. For example, the phrase “Fluoride saves lives—said no one ever” could be misclassified due to the absence of tone recognition (Gandy et al. 2025). Our preprocessing pipeline, while effective in removing noise, did not account for rhetorical or figurative language, which may have influenced classification outcomes.

While this study employed a basic polarity schema (positive, neutral, negative), we acknowledge that such surface-level classification may not capture deeper motivations or themes behind sentiment expression (Umunyana et al. 2024). Posts related to CWF often reflect nuanced concerns—such as distrust in authorities, child safety, or environmental risks—that cannot be fully understood through a simple polarity. Future studies should consider integrating named entity recognition, thematic co-occurrence mapping, or qualitative coding to enrich the interpretive depth of sentiment analysis in public health contexts.

While transformer-based models such as BERT or RoBERTa offer significantly improved contextual awareness and language nuance detection (Mohammad et al. 2024), their use was not feasible in this study due to computational constraints and the need to preserve model interpretability for trend-level analysis. Traditional models used here, such as logistic regression and random forest, rely on bag-of-words and term frequency–inverse document frequency (TF-IDF) features, which cannot capture word order or semantic context, limiting their effectiveness for interpreting informal or complex social media language (Alkhnbashi et al. 2024). Future studies may consider using domain-adapted transformer architectures, such as RoBERTa-Large or BERTweet, to enhance sentiment granularity and detect more subtle expressions of doubt, sarcasm, or misinformation.

The study has several limitations. Text-only analysis excluded images and videos, which are commonly used on platforms such as Instagram and may convey strong emotional cues (Chun 2024). This focus on text was due to practical constraints: Meta’s API offers more reliable access to text, our NLP tools are not designed for multimedia inputs, and multimodal analysis requires distinct infrastructure. Posts were treated equally in the study, without weighting based on user engagement. This may have reduced the perceived impact of highly shared or liked content. Although engagement statistics were recorded, the relationship between sentiment and engagement was not explored, despite emotional content often receiving greater interaction (Majó‑Vázquez et al. 2020).

The dataset included only English-language posts, limiting the representation of multilingual or culturally diverse perspectives (Boon-Itt and Skunkan 2020). Social media users do not represent the general population; individuals such as older adults, rural residents, and those with limited internet access are often underrepresented. Moreover, many users may choose not to express their views or avoid specific topics altogether. Content that is emotionally charged or controversial is more likely to be amplified through likes and shares, resulting in increased engagement and potential selection bias. This can result in a dataset that overrepresents polarized or negative sentiment, potentially misrepresenting broader community perspectives (Wang et al. 2019; Cinelli et al. 2020).

The credibility of content sources was not assessed. Distinguishing posts from health authorities, influencers, bots, or anonymous users could help trace the origins and spread of misinformation. Future studies should investigate whether negative posts receive more shares or neutral posts receive more comments and incorporate source verification across platforms such as TikTok and YouTube to understand content dynamics better.

Overall, this study provides valuable insights into the nature of online discussions related to CWF sentiment and highlights the impact of social media on public health discussions. Although these platforms enable wide-reaching engagement, they also facilitate the rapid spread of misinformation. Addressing this challenge requires a combination of real-time monitoring, improved analytical tools, and transparent communication strategies. Integrating more advanced sentiment analysis techniques into public health systems may support the earlier identification of sentiment shifts and help maintain trust in evidence-based health interventions.

## Author Contributions

N. Torwane, contributed to conception and design, data interpretation, drafted and critically revised the manuscript; R. Lalloo, contributed to conception and design, data interpretation, drafted and critically revised the manuscript; D. Ha, contributed to conception and design, data interpretation, drafted critically revised the manuscript; L. Do, contributed to conception and design, drafted and critically revised the manuscript. All authors gave final approval and agree to be accountable for all aspects of the work.

## Supplemental Material

sj-docx-1-jct-10.1177_23800844251369336 – Supplemental material for Analysis of Water Fluoridation Debates on Meta Platforms Using Advanced Machine Learning Approaches Supplemental material, sj-docx-1-jct-10.1177_23800844251369336 for Analysis of Water Fluoridation Debates on Meta Platforms Using Advanced Machine Learning Approaches by N. Torwane, R. Lalloo, D. Ha and L. Do in JDR Clinical & Translational Research

sj-docx-2-jct-10.1177_23800844251369336 – Supplemental material for Analysis of Water Fluoridation Debates on Meta Platforms Using Advanced Machine Learning Approaches Supplemental material, sj-docx-2-jct-10.1177_23800844251369336 for Analysis of Water Fluoridation Debates on Meta Platforms Using Advanced Machine Learning Approaches by N. Torwane, R. Lalloo, D. Ha and L. Do in JDR Clinical & Translational Research

## References

[bibr1-23800844251369336] Black DR Dietz JE Stirratt AA Coster DC . 2015. Do social media have a place in public health emergency response? J Emerg Manag. 13(3):217–226. 26150365 10.5055/jem.2015.0235

[bibr2-23800844251369336] Boon-Itt S Skunkan Y. 2020. Public perception of the COVID-19 pandemic on Twitter: sentiment analysis and topic modelling study. JMIR Public Health Surveill. 6(4):e21978. 10.2196/21978. PMC7661106 33108310

[bibr3-23800844251369336] Breiman L. 2001. Random forests. Mach Learn. 45(1):5–32.

[bibr4-23800844251369336] Canadian Academy of Health Sciences. 2014. Improving access to oral health care for vulnerable people living in Canada. Ottawa (Canada): CAHS.

[bibr5-23800844251369336] Cavazos-Rehg PA Krauss M Fisher SL Salyer P Grucza RA Bierut LJ . 2015. Twitter chatter about marijuana. J Adolesc Health. 56(2):139–145. 25620299 10.1016/j.jadohealth.2014.10.270 PMC4306811

[bibr6-23800844251369336] Cheng J Bernstein M Danescu-Niculescu-Mizil C Leskovec J. 2017. Anyone can become a troll: causes of trolling behavior in online discussions. CSCW Conf Comput Support Coop Work. 2017:1217–1230. 29399664 10.1145/2998181.2998213 PMC5791909

[bibr7-23800844251369336] Chou WS Oh A Klein WMP . 2018. Addressing health-related misinformation on social media. JAMA. 320(23):2417–2418. 30428002 10.1001/jama.2018.16865

[bibr8-23800844251369336] Chun J. 2024. MultiSentimentArcs: a novel method to measure coherence in multimodal sentiment analysis for long-form narratives in film. Front Comput Sci. 6:1444549. 10.3389/fcomp.2024.1444549

[bibr9-23800844251369336] Cinelli M Quattrociocchi W Galeazzi A Valensise CM Brugnoli E Schmidt AL Zola P Zollo F Scala A. 2020. The COVID-19 social media infodemic. Sci Rep. 10(1):16598. 10.1038/s41598-020-73510-5. 33024152 PMC7538912

[bibr10-23800844251369336] City of Calgary. 2021. 2021 results—Calgary general election. Calgary (Canada): Elections Calgary; [accessed 2025 Jul 22] https://www.calgary.ca/election/results/2021-results.html.

[bibr11-23800844251369336] Eysenbach G. 2011. Infodemiology and infoveillance: tracking online health information and cyberbehavior for public health. Am J Prev Med. 40(5 suppl 2):S154–S158. 10.1016/j.amepre.2011.02.006 21521589

[bibr12-23800844251369336] Gabarron E Wynn R. 2016. Use of social media for sexual health promotion: a scoping review. Glob Health Action. 9(1):32193. 10.3402/gha.v9.32193. 27649758 PMC5030258

[bibr13-23800844251369336] Gandy LM Ivanitskaya LV Bacon LL Bizri-Baryak R. 2025. Public health discussions on social media: evaluating automated sentiment analysis methods. JMIR Form Res. 9:e57395. 10.2196/57395. PMC11784633 39773420

[bibr14-23800844251369336] George DR Rovniak LS Kraschnewski JL . 2013. Dangers and opportunities for social media in medicine. Clin Obstet Gynecol. 56(3):453–462. 23903375 10.1097/GRF.0b013e318297dc38 PMC3863578

[bibr15-23800844251369336] Helmi M Spinella MK Seymour B. 2018. Community water fluoridation online: an analysis of the digital media ecosystem. J Public Health Dent. 78(4):296–305. 29603251 10.1111/jphd.12268

[bibr16-23800844251369336] Hong T Tang Z Lu M Wang Y Wu J Wijaya D. 2023. Effects of #coronavirus content moderation on misinformation and anti-Asian hate on Instagram. New Media Soc. 27(2):931–954.

[bibr17-23800844251369336] Hutto CJ Gilbert E. 2014. VADER: a parsimonious rule-based model for sentiment analysis of social media text. Proceedings of the Eighth International AAAI Conference on Weblogs and Social Media. 8(1):216–225.

[bibr18-23800844251369336] Iheozor-Ejiofor Z Walsh T Lewis SR Riley P Boyers D Clarkson JE Worthington HV Glenny AM O’Malley L. 2024. Water fluoridation for the prevention of dental caries. Cochrane Database Syst Rev. 10(10):CD010856. 10.1002/14651858.CD010856.pub3. PMC11449566 39362658

[bibr19-23800844251369336] James G Witten D Hastie T Tibshirani R. 2013. An introduction to statistical learning: with applications in R. 1st ed. New York: Springer.

[bibr20-23800844251369336] Larson HJ de Figueiredo A Xiahong Z Schulz WS Verger P Johnston IG Cook AR Jones NS. 2016. The state of vaccine confidence 2016: global insights through a 67-country survey. EBioMedicine. 12:295–301. 10.1016/j.ebiom.2016.08.042. 27658738 PMC5078590

[bibr21-23800844251369336] Lotto M Menezes TS Zakir Hussain I Tsao S Butt ZA Morita PP Cruvinel T. 2022. Characterization of false or misleading fluoride content on Instagram: infodemiology study. J Med Internet Res. 24(5):e37519. 10.2196/37519. PMC9164089 35588055

[bibr22-23800844251369336] Mackert M Bouchacourt L Lazard A Wilcox GB Kemp D Kahlor LA George C Stewart B Wolfe J. 2021. Social media conversations about community water fluoridation: formative research to guide health communication. J Public Health Dent. 81(2):162–166. 33058200 10.1111/jphd.12404

[bibr23-23800844251369336] Majó‑Vázquez S Nielsen RK Verdú J Rao DN De Domenico M Papaspiliopoulos O. 2020. Volume and patterns of toxicity in social media conversations during the COVID‑19 pandemic. Oxford (UK): Reuters Institute for the Study of Journalism. 10.60625/risj-5902-2323.

[bibr24-23800844251369336] McLaren L Patterson S Thawer S Faris P McNeil D Potestio M Shwart L. 2016. Measuring the short-term impact of fluoridation cessation on dental caries in grade 2 children using tooth surface indices. Community Dent Oral Epidemiol. 44(3):274–282. 26888380 10.1111/cdoe.12215 PMC5021129

[bibr25-23800844251369336] McLaren L Singhal S. 2016. Does cessation of community water fluoridation lead to an increase in tooth decay? A systematic review of published studies. J Epidemiol Community Health. 70(9):934–940. 27177581 10.1136/jech-2015-206502 PMC5013153

[bibr26-23800844251369336] Meier S Stock C Krämer A. 2007. The contribution of health discussion groups with students to campus health promotion. Health Promot Int. 22(1):28–36. 17164269 10.1093/heapro/dal041

[bibr27-23800844251369336] Mertz A Allukian M. 2014. Community water fluoridation on the internet and social media. J Mass Dent Soc. 63(2):32–36. 25230407

[bibr28-23800844251369336] Mohammad R. Alkhnbashi O. S. Hammoudeh M. (2024). Optimizing Large Language Models for Arabic Healthcare Communication: A Focus on Patient-Centered NLP Applications. Big Data and Cognitive Computing, 8(11): 1–26. Article 157. 10.3390/bdcc8110157

[bibr29-23800844251369336] Nguyen A Catalan-Matamoros D. 2022. Anti-vaccine discourse on social media: an exploratory audit of negative tweets about vaccines and their posters. Vaccines. 10(12):2067. 10.3390/vaccines10122067 PMC9782243 36560477

[bibr30-23800844251369336] O’Neill B Kapoor T McLaren L. 2019. Politics, science, and termination: a case study of water fluoridation policy in Calgary in 2011. Rev Policy Res. 36(1):99–120.

[bibr31-23800844251369336] Oh HJ Kim CH Jeon JG . 2020. Public sense of water fluoridation as reflected on Twitter 2009–2017. J Dent Res. 99(1):11–17. 31682777 10.1177/0022034519885610

[bibr32-23800844251369336] Quinlan JR. 1986. Induction of decision trees. Mach Learn. 1(1):81–106.

[bibr33-23800844251369336] Roozenbeek J van der Linden S. 2020. Fake news game confers psychological resistance against online misinformation. Palgrave Commun. 5:65. 10.1057/s41599-019-0279-9.

[bibr34-23800844251369336] Statista Research Department. 2024. Facebook: number of monthly active users worldwide 2008–2024. Hamburg (Germany): Statista; [accessed 2024 Dec 24]. https://www.statista.com/statistics/264810/number-of-monthly-active-facebook-users-worldwide/.

[bibr35-23800844251369336] Taddi VV Kohli RK Puri P. 2024. Perception, use of social media, and its impact on the mental health of Indian adolescents: a qualitative study. World J Clin Pediatr. 13(3):97501. 10.5409/wjcp.v13.i3.97501. 39350908 PMC11438920

[bibr36-23800844251369336] Umunyana C Tuyizere G Mbarushimana A. 2024. Students’ sentiment analysis using natural language toolkit in machine learning for module evaluation. Eur J Eng Technol Res. 9(1):72–75.

[bibr37-23800844251369336] Vosoughi S Roy D Aral S. 2018. The spread of true and false news online. Science. 359(6380):1146–1151. 29590045 10.1126/science.aap9559

[bibr38-23800844251369336] Su V Thakur N. 2025. COVID-19 on YouTube: a data-driven analysis of sentiment, toxicity, and content recommendations. 2025 IEEE 15th Annual Computing and Communication Workshop and Conference (CCWC); 2025 Jan 6-8; Las Vegas, NV, USA. pp. 00715-00723. Piscataway (NJ): IEEE. 10.1109/CCWC62904.2025.10903713.

