# Supplementary material for: Analysis of Water Fluoridation Debates on Meta Platforms Using Advanced Machine Learning Approaches
Source: JDR Clin Trans Res. 2025 Sep 15;11(3):454–64. doi: 10.1177/23800844251369336 (PMC13232570; doi:10.1177/23800844251369336)
Supplement: Supplementary file 2 [file sj-docx-2-jct-10.1177_23800844251369336.docx]

STROBE Statement-

(Checklist of items that should be included in reports of cross-sectional studies)

| **Item No** | **Recommendation** | **Location in Manuscript** |
| --- | --- | --- |
| 1 | (a) Indicate the study’s design with a commonly used term in the title or the abstract (b) Provide in the abstract an informative and balanced summary of what was done and what was found | Title page; Abstract |
| 2 | Explain the scientific background and rationale for the investigation being reported | Introduction – Paragraphs 1–2 |
| 3 | State specific objectives, including any prespecified hypotheses | Introduction – Final paragraph |
| 4 | Present key elements of study design early in the paper | Methods – Scope of the Study |
| 5 | Describe the setting, locations, and relevant dates, including periods of recruitment, exposure, follow-up, and data collection | Methods – Scope of the Study and Data Collection |
| 6 | (a) Give the eligibility criteria, and the sources and methods of selection of participants | Not applicable – public social media data used |
| 7 | Clearly define all outcomes, exposures, predictors, potential confounders, and effect modifiers | Methods – Sentiment Classification Framework and Factor-Based Analysis |
| 8 | For each variable of interest, give sources of data and details of methods of assessment | Methods – Data Collection and Sentiment Classification Framework |
| 9 | Describe any efforts to address potential sources of bias | Methods – Ethical Approval and Data Confidentiality |
| 10 | Explain how the study size was arrived at | Methods – Scope of the Study |
| 11 | Explain how quantitative variables were handled in the analyses | Methods – Sentiment Scoring and Engagement Metrics Analysis |
| 12 | (a-e) Describe all statistical methods | Methods – Model Validation and Performance Evaluation |
| 13 | (a-c) Report numbers of individuals at each stage of study | Results – Engagement Metrics Analyses |
| 14 | (a-b) Give characteristics of study participants | Not applicable – aggregate-level public post data |
| 15 | Report numbers of outcome events or summary measures | Results – Sentiment Trends and Sentiment Categories |
| 16 | (a-c) Give unadjusted and adjusted estimates | Results – Sentiment Model Evaluation Table |
| 17 | Report other analyses done | Results – Word Sentiment and Co-occurrence Network Analysis |
| 18 | Summarise key results with reference to study objectives | Discussion – Opening paragraph |
| 19 | Discuss limitations of the study | Discussion – Limitations section |
| 20 | Give a cautious overall interpretation of results | Discussion – Interpretation section |
| 21 | Discuss the generalisability of the study results | Discussion – Final paragraphs |
| 22 | Give the source of funding and role of funders | Acknowledgements and Funding Declaration |
